# Older Adults’ Perspectives on Artificial Intelligence as a Source of Advice

**DOI:** 10.1093/geroni/igaf122.1371

**Published:** 2025-12-31

**Authors:** Lauren Cerino, Adam Felts, Alexa Balmuth, Taylor Brennan, Chaiwoo Lee, Joseph Coughlin

**Affiliations:** Massachusetts Institute of Technology, Cambridge, Massachusetts, United States; Massachusetts Institute of Technology, Cambridge, Massachusetts, United States; Massachusetts Institute of Technology, Cambridge, Massachusetts, United States; MIT, Boston College, Cambridge, Massachusetts, United States; Massachusetts Institute of Technology, Cambridge, Massachusetts, United States; Massachusetts Institute of Technology AgeLab, Cambridge, Massachusetts, United States

## Abstract

A large proportion of Americans use generative AI tools to seek information and advice. Prior research suggests that older people are less likely than their younger counterparts to use generative AI (NORC, 2023), but older adults’ attitudes toward AI technologies remain relatively obscure. Drawing on data from a national online survey study of American adults between ages 18 to 96 (N = 1008), this study examines older adults’ experiences with AI and openness to receiving advice from AI-enabled tools. The survey included questions about experiences with AI and using AI-enabled chatbots for general use and advice. Preliminary findings indicate that most older adults had some level of familiarity with AI-enabled technologies, with nearly three-quarters (73.7%) of respondents aged 60 and older at least “a little” familiar. However, fewer (28.3%) in this age group reported having used AI-enabled technology. Older adults exhibited greater skepticism toward AI-generated advice than younger respondents; compared to younger age groups, fewer older respondents (36.0%) considered AI to be a valid source of advice, and 34.7% of older respondents indicated that they did not at all trust AI-enabled tools at all to give good advice. Mean trust scores also declined with age. These findings contribute to understanding about attitudes toward and adoption of AI across age groups. As generative AI becomes an increasingly common presence in everyday life, knowing how different generations engage with these tools is essential for understanding how they may be adopted, the extent of their disruptive potential, and the emergences of disparities of access.

